# Photobase effect for just-in-time delivery in photocatalytic hydrogen generation

**DOI:** 10.1038/s41467-020-18583-6

**Published:** 2020-10-14

**Authors:** Jiawen Fang, Tushar Debnath, Santanu Bhattacharyya, Markus Döblinger, Jochen Feldmann, Jacek K. Stolarczyk

**Affiliations:** 1 Chair for Photonics and Optoelectronics, Nano-Institute Munich, Department of Physics, Ludwig-Maximilians-Universität (LMU), Königinstr. 10, 80539 Munich, Germany; 2 Department of Chemistry, Ludwig-Maximilians-Universität München, Butenandtstr. 5–13 (E), 81377 Munich, Germany; 3 Present Address: Department of Chemical Sciences, IISER Berhampur, Transit Campus (Govt. ITI Building), Engg. School Junction, Berhampur, Odisha 760010 India

**Keywords:** Energy, Photocatalysis, Nanoparticles, Surfaces, interfaces and thin films

## Abstract

Carbon dots (CDs) are a promising nanomaterial for photocatalytic applications. However, the mechanism of the photocatalytic processes remains the subject of a debate due to the complex internal structure of the CDs, comprising crystalline and molecular units embedded in an amorphous matrix, rendering the analysis of the charge and energy transfer pathways between the constituent parts very challenging. Here we propose that the photobasic effect, that is the abstraction of a proton from water upon excitation by light, facilitates the photoexcited electron transfer to the proton. We show that the controlled inclusion in CDs of a model photobase, acridine, resembling the molecular moieties found in photocatalytically active CDs, strongly increases hydrogen generation. Ultrafast spectroscopy measurements reveal proton transfer within 30 ps of the excitation. This way, we use a model system to show that the photobasic effect may be contributing to the photocatalytic H_2_ generation of carbon nanomaterials and suggest that it may be tuned to achieve further improvements. The study demonstrates the critical role of the understanding the dynamics of the CDs in the design of next generation photocatalysts.

## Abstract (web summary)

Efficient photocatalytic hydrogen generation relies on highly choreographed delivery of protons and electrons to the reaction site such that they arrive just in time. Here, authors show that proton transport can be controlled by light with the photobase effect to increase H_2_ generation rate.

## Introduction

In photocatalytic hydrogen generation the energy of an incident photon is utilized to drive the energetically up-hill water splitting reaction^1,2^. The evolved hydrogen can then serve directly as an energy-rich fuel or as a reactant in chemical synthesis of carbon-based fuels, e.g. methane in the Sabatier or Fischer-Tropsch processes^3,4^. This approach is an appealing alternative to harvest and store abundant solar energy, potentially providing a renewable solution for the global energy supply^5^. In general, the photocatalytic reaction involves a complex sequence of charge carrier (electron and hole) generation, separation and transfer steps, and typically requires a concurrent proton transfer. The efficiency of such proton-coupled electron transfer (PCET) processes is limited by its slowest component, which could be reactant diffusion^6^. Consequently, a major challenge in photocatalytic H_2_ production is to ensure that all participants in the process (photoexcited charge carriers, protons, and possibly other molecules) are delivered to the reaction site at optimal time. Otherwise, the photoexcited carriers may recombine, photodegrade the catalysts or induce unwanted side reactions, in all cases reducing the activity.

Photobases are molecules that have higher proton affinity in the excited state than in the ground state. In other words, the pK_a_ in the excited state is much higher than in the ground state, so that upon photoexcitation the now stronger base abstracts a proton from the environment. Hence, such molecules offer an opportunity to control the proton transfer by light^7,8^. For instance, quinoline photobases have been shown to form protonated species within tens of picoseconds of the light excitation^9^. Effectively, the photobase can ensure that the proton promptly and selectively arrives at the reduction site. This result can also be achieved by other means, for example by lowering the pH. Nonetheless, utilizing the excited state acid-base equilibria the same effect can conceivably be realized in a neutral medium, without the potentially corrosive acidic conditions. Therefore, for photocatalytic H_2_ generation the photobases present an appealing approach, wherein the incident photons not only provide the energy for the water splitting, but also dynamically alter the photocatalyst to enhance the process efficiency^10^.

Nanostructured photocatalysts, either inorganic, organic, or hybrid, rose to the forefront of the photocatalytic field because their composition, structure, and surface can be controlled to tailor the optoelectronic and morphological properties to the desired function^11–13^. Carbon dots (CDs) are a recent entrant into photocatalysis that has attracted attention due to their versatility, photostability, absence of heavy metals, ease of preparation, and tunable properties^14,15^. This flexibility derives from their complex internal structure, inherent disorder, and surface functionality. Effectively, small changes in the preparative procedure can result in large differences in the properties. While the application possibilities are impressive, there is still no detailed understanding of their structure and the mechanism of photocatalytic reactions^16–18^. They are generally considered to consist of aromatic, *sp*^*2*^-hybridized domains immersed in a *sp*^*3*^-hybridized amorphous matrix with various functional groups on the surface^19,20^. In this context, we have shown that the optical properties of CDs prepared from citric acid and ethylenediamine can be reproduced by a simple model of polycyclic hydrocarbons embedded in an amorphous polymer, poly(methyl methacrylate)^20^. Heterocyclic compounds, including strong molecular fluorophores, form in the presence of nitrogen-containing precursors^21,22^. Importantly, the position of the nitrogen atom dopant in the heterocyclic aromatic structure, controlled by the synthetic procedure, determines the functionality of the CDs. Whilst the graphitic nitrogen increases the photoluminescence (PL) quantum yield, pyridinic and pyrrolic nitrogen yields much higher photocatalytic H_2_ generation activity^23^. Pyridine and its larger analogs, such as quinoline or acridine, are weak bases that can be protonated at the nitrogen atom in the excited state, suggesting that such photocatalytically active CDs contain photobasic moieties in their structure. Interestingly, heptazine (tri-*s*-triazine), the building block of graphitic carbon nitrides (g-CN), also contains multiple N atoms at the edge sites of the aromatic structure and exhibits photobasic behavior^24,25^. Recent calculations show that in the excited state heptazine molecules induce proton and electron transfer from hydrogen-bonded water molecules leading to heptazinyl radicals^26,27^. These species can undergo further photolysis, recovering the heptazine and releasing hydrogen radicals that further react to form molecular H_2_. In corroborating evidence, Electron Paramagnetic Resonance (EPR) experiments show that a long-lived radical forms in cyanamide-functionalized heptazine-based g-CN upon illumination that can be later, under dark conditions, used to produce H_2_^28–30^. It has been observed that protonation of the g-CN structure in acidic media increases the H_2_ evolution rate^31–33^. This further supports the argument that the proton transfer rate may be a limiting factor in the photocatalytic H_2_ generation on g-CN. Overall, these experimental and computational results offer tantalizing hints that the photobasic effect of the constituent units in CDs and in g-CN is highly beneficial for the photocatalytic activity of these nanomaterials and at least partially responsible for their impressive H_2_ formation rates.

In this paper, we show that the introduction of photobasic units into carbon dots indeed increases the H_2_ generation rate, on the basis of time-resolved spectroscopy and photocatalytic activity measurements. Due to complexity of nitrogen-containing CDs, it is difficult to find an appropriate blank reference sample which exhibits no nitrogen-related acid-base activity. Therefore, we started with polyethylene glycol (PEG) derived nitrogen-free CDs and introduced acridine, a model photobase, into the photocatalyst. Acridine is an *N*-heterocyclic aromatic compound, resembling molecules likely present in the CDs^20^, with pK_a_ = 5.5 in the ground state and pK_a_* = 10.7 in the excited state^34–36^. This means that at pH 7 it is protonated to a very small extent (3%), but becomes mostly protonated in the excited state (see details in the Supplementary Note 1). Calculations suggest that excitation of acridine in water induces proton-coupled electron transfer leading to acridinyl radical that can split under illumination to release a hydrogen radical^37^. A common acridine-based derivative photobase, acridine orange, can abstract a proton from alcohols coupled with an electron transfer that can also result in acridinyl-based radicals^38,39^. This further implied that acridine in its excited protonated state is prone to further reduction reactions (e.g. accepting an electron), making it suitable for studying H_2_ generation from water^10^. These studies were focused on molecular interactions of acridine with a potential proton and electron donor (alcohol and water). In our work where acridine is integrated with CDs the process can be even more efficient. This is because the electron can also be readily transferred from the photoexcited CDs, which have a broader absorption range.

In short, we synthesize CDs which comprise a model photobase and we show that the photobasic effect can be a plausible element of the mechanism of photocatalytic hydrogen generation with CDs. The understanding of the mechanism demonstrates that careful design of the CDs enables control by light of the proton transfer rates at the photocatalyst surface to enhance the efficiency of H_2_ production. In this way, we believe it opens a new avenue for modifying the functionality of CDs to bring further improvements in efficiency.

## Results

### Preparation and characterization of CDs

**Fig. 1:**
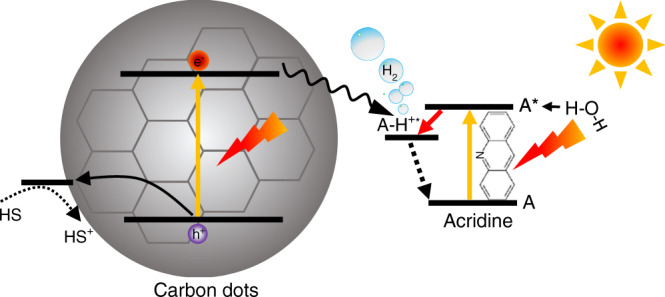
Photobase effect. Schematic of the H_2_ generation on the CD-acridine photocatalyst in which, upon light excitation, acridine (A) abstracts a proton from water.

**Fig. 2:**
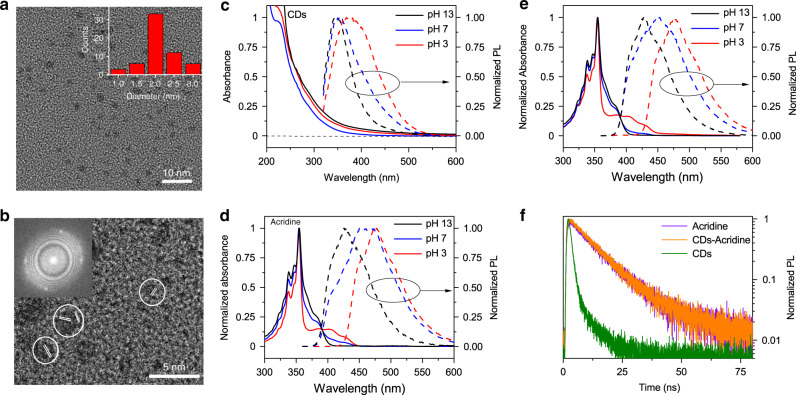
Morphological and optical properties of CDs. **a** TEM image of CDs (size distribution in inset); **b** HR-TEM image of CDs (FFT image in inset). UV-Vis absorption (solid lines) and photoluminescence (dashed lines) spectra of **c** CDs, **d** acridine, and **e** CD-acridine at different pH values. **f** Time-resolved PL decay of CDs, acridine, and CD-acridine, measured at 450 nm. For the acquisition of PL, in all cases the samples were excited at 350 nm.

The nitrogen-free CDs were prepared in the first step and then combined with a model photobase, acridine (cf. Fig. 1), to demonstrate an increase in photocatalytic activity. The CDs were synthesized in an autoclave from PEG dissolved in ethanol by pyrolysis at 200 °C^40^. The details of the preparation are provided in the Methods sections. Following the synthesis, ethanol was evaporated and the CDs were dispersed in water. Transmission electron microscopy (TEM, see Fig. 2a and Supplementary Fig. 1) and dynamic light scattering (Supplementary Fig. 2) measurements reveal the formation of ultrasmall monodisperse particles 2 nm in diameter (inset in Fig. 2a). Upon close inspection, most particles exhibit crystal fringes, with lattice plane distances ~2.35 Å, indicating an at least partially crystalline structure (Fig. 2b)^23,41^.

In the next step, the optical properties of the CDs were investigated. As the acid-base equilibrium is determined by the pH of the medium, we varied the proton concentration. We focused on three values of pH: 3, 7, and 13, chosen to represent the three relevant populations of acridine: always protonated (pH 3, i.e. acridinium cation, Acr-H^+^), mostly non-protonated in the ground state, but protonated to a larger extent in the excited state (pH 7), and always non-protonated (pH 13), consistently with the excited state populations reported from fluorescence time-resolved studies^42^. In effect, these pH values are on either side of acridine pK_a_ and pK_a_* (5.5 and 10.7, respectively). For reference purposes, the same values were used also for the nitrogen-free CDs. The absorption spectra of the CDs, presented in Fig. 2c, do not exhibit any significant dependence on pH. They are nearly featureless, but contain a long absorption tail extending weakly into the visible part of the spectrum. The CDs are photoluminescent at all pH values, with a single PL peak in every spectrum. However, the Stokes shift is pH-dependent, with the PL maxima at between 344 nm (pH 13) and 375 nm (pH 3). The dependence of PL on proton concentration is in agreement with literature and can be attributed to the presence of hydroxyl groups on the surface of the CDs^43^. The Fig. 2d shows the absorption and PL spectra of the second component of the photocatalyst, i.e. of acridine. The sharp absorption peaks at 354 nm can be ascribed to transitions to singlet state S_2_, whose energy is largely unaffected by the changes in pH^35,44^. This absorption range is very convenient for the excitation during the optical spectroscopy experiments. In contrast to S_2_, the energy level of S_1_ state is lower in the protonated state, leading to a pronounced red-shift at pH 3 of the secondary peak at longer wavelengths^35^. As mentioned before, acridine is mostly non-protonated at pH 7. Hence, the normalized absorption spectrum in neutral conditions is identical to the spectrum acquired at pH 13, with the exception of a small peak at 430 nm due to a small fraction of the protonated species. The PL quantum yield of acridine is strongly dependent on pH, with the yield decreasing with pH^35,45^. The spectra are also different with the peak of emission of the protonated species (pH 3) at 475 nm and of the non-protonated species (pH 13) at 427 nm. The photobasic behavior of acridine manifests itself in the PL of the sample at pH 7, which comprises the emission peaks of both species. In comparison to absorption, where the spectra of samples at pH 7 and pH 13 are nearly identical, PL emission at pH 7 appears red-shifted. The non-normalized emission spectra (see Supplementary Fig. 3) prove that, despite the higher PL quantum yield of protonated acridine, the small fraction (3%) of the protonated species at pH 7 cannot account for the shift alone. Hence, it is clear that a substantial part of acridine molecules become protonated in the excited state before the radiative charge recombination. This is confirmed by measurements of weakened PL emission of acridine in media of higher pK_a_ (ethanol, 15.6) and increased intensity in media of lower pK_a_ (NH_4_Cl solution, pK_a_ = 9.2 and boric acid solution, pK_a_ = 9.2), shown in Supplementary Fig. 4.

The combined CD-acridine samples were prepared in two ways. In one method, the CDs were synthesized first, transferred to water, and mixed with an aqueous solution of acridine. This approach was designed to adsorb acridine on the surface of the CDs. In the second method, acridine (same amount as above) was mixed with the precursor of the CDs, PEG, and the synthetic procedure was carried out as before. After the carbonization, acridine could be located within the CDs either as free molecules or incorporated into larger N-hetorocyclic domains, mimicking the proposed composition and structure of N-doped CDs prepared from citric acid and diamines^20^. Due to different modes of acridine inclusion in this method, complicating the understanding of individual contributions, we used the second method as reference samples only, focusing in the further analysis on the first method that leads to CDs with acridine adsorbed on the outside. The absorption and PL spectra of this CD-acridine sample (see Fig. 2e) show strong resemblance to the free acridine sample. The comparison of non-normalized absorption spectra of CD-acridine with its individual components (see Supplementary Fig. 5) confirms a dominant contribution of acridine in the range 325–400 nm, but a significant contribution of CDs for shorter and longer wavelengths where acridine does not absorb. To investigate the role of the PL emission from the CDs in this combined system, we acquired the PL excitation (PLE) spectrum at 355 nm (see Supplementary Fig. 6). This wavelength was chosen to match the free CDs emission (cf. Fig. 2c). Indeed, the PLE spectrum shows the emission originating at 286 nm, corresponding to the absorption by the aromatic structures in the CDs. Interestingly, in the combined sample, the PL at 350 nm is quenched (cf. Supplementary Fig. 7), suggesting energy or electron transfer from CDs to acridine. The Forster energy transfer pathway appears possible because the 350 nm is very close to the peak acridine absorption. Hence, in a further experiment, we measured the PLE spectra setting the detection wavelength to 450 nm, i.e., at peak acridine emission. As shown in Supplementary Fig. 8, the PLE of the CD-acridine system is higher than of acridine alone in the region 280–320 nm that coincides with strong CD absorption. The emission of CDs at 450 nm is weak, implying that indeed the energy transfer from CDs to acridine can take place. Although the time-correlated single photon counting (TCSPC) measurements at 450 nm (Fig. 2f) show that the PL decay dynamics of the CD-acridine sample is the same as in free acridine, this does not contradict the electron transfer, because the native CD lifetime is shorter than of acridine. To verify the possibility of electron transfer, we performed cyclic voltammetry measurements of CDs and acridine in mildly alkaline conditions to determine the relative positions of the conduction band edge of the CDs versus the LUMO level of acridine. In the voltammograms these levels are manifested as cathodic (reduction) peaks. Supplementary Figure 9 reveals that there are prominent cathodic peaks at around −1.03 V and −0.92 V vs Ag/AgCl for CDs and acridine, respectively. Assuming that no significant reorganization occurs in the excited state of CDs, the reduction potential in the ground state gives a good representation of the oxidation potential in the excited state. Hence, the voltammetry results imply ~100 meV difference in the energy levels that enables electron transfer from excited CDs to the LUMO of non-protonated acridine. Importantly, this difference is even larger in the acidic conditions because the LUMO level of protonated acridine is lower, providing a stronger driving force^36,46^. Overall, these measurement suggest that both energy and electron transfers can take place. The second important conclusion to be drawn from the PL measurements of the combined system is that the photobasic properties of acridine should be similar in both free acridine and in the CD-acridine sample. This means that free acridine can be treated as a good starting point for the investigation of the charge carrier and proton transfer dynamics.

### Proton transfer rate

**Fig. 3:**
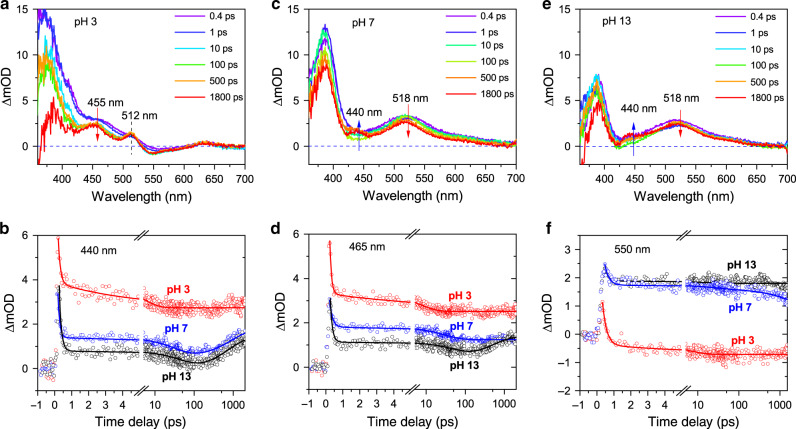
Protonation dynamics. Transient absorption spectra of acridine at **a** pH 3, **c** pH 7, and **e** pH 13. Comparison of the transient absorption traces at **b** 440 nm, **d** 465 nm, and **f** 550 nm at pH 3, pH 7, and pH 13. Open circles represent the data points, the numerical fits are plotted as the solid lines.

Transient absorption spectroscopy was then used to determine the rate of proton transfer in the excited state of acridine-based systems. To this end, the acridine solutions in water at different pH values were excited with 100 fs pump pulses at 350 nm and the differential absorption spectra were acquired with a broad band pulse after a time delay up to 2.0 ns (see Fig. 3). The spectrum at pH 3 contains two excited state absorption (ESA) maxima, at 455 nm and 512 nm, respectively. The intersystem crossing rate is considered to be very low in the acridinium cation^9^, therefore the peaks are attributed to excitations from S_1_ to S_n_ for the protonated and non-protonated species, respectively^47^. The singlet spin multiplicity of the molecules is corroborated by the presence of a negative ΔOD region at 530–570 nm. There is no absorption of the protonated species in this region, but it overlaps with the low-energy tail of the PL emission. Therefore, it can be ascribed to stimulated emission of the singlet Acr-H^+^. The presence of the minor ESA peak at 512 nm indicates a presence of a small fraction of non-protonated acridine even at pH 3. This assignment can be inferred from the position of the corresponding peak observed at 518 nm at pH 7 and 13 (Fig. 3c, e), with the shift caused by the overlap with the strong emission of Acr-H+ in acidic conditions. To ascertain the claim, we performed an analogous experiment at lower pH, where the non-protonated acridine would not be expected at all. Indeed, as shown in Supplementary Fig. 10, the peak vanishes completely at pH 1 and the broad stimulated emission has an onset already at 520 nm, confirming the assignment. Meanwhile, at alkaline conditions (Fig. 3e), the PL is much weaker and no stimulated emission regions can be seen. This is in agreement with a faster intersystem crossing and low PL quantum yield of the S_1_ state expected in the non-protonated species^35,36,44^. The resulting higher triplet state then relaxes to lower lying T_1_ state^36^. The two ESA peaks at 518 nm and 440 nm are then attributed to the excitation from the S_1_ to S_n_ and from T_1_ to T_n_, respectively (see Supplementary Fig. 11)^47^. No traces of protonation are observed, as expected at this pH. This was also confirmed by taking the measurements in ethanol which is harder to deprotonate than water (see Supplementary Fig. 12). The differential spectrum in ethanol contains the same ESA peaks with a very clear conversion between then, consistent with a transition from S_1_ to T_1_. The same process, albeit on a smaller scale, is also visible in water. The differential spectra at pH 7 (see Fig. 3c) are broadly similar to the ones acquired at pH 13, which is in agreement with the mostly deprotonated state of acridine at these conditions.

To inspect the dynamics of system more closely, the transient absorption traces of the acridine were plotted in Fig. 3 for several relevant wavelengths. The aim was to detect the formation of the protonated species, expected ~450 nm (cf. Fig. 3a). However, at 440 nm the traces are essentially the same at pH 7 and pH 13, in contrast to pH 3. This is because the signal is then dominated by the rise, after ~65 ps, of the ESA peak corresponding to T_1_ to T_n_ transition of non-protonated acridine. This rise is absent in acidic conditions. To shift away from this ESA peak, in Fig. 3d we plotted the traces recorded at 465 nm. Here it becomes clear, that the signal at pH 7 follows the dynamics of the non-protonated acridine until 30 ps (cf. Supplementary Table 2), when the two traces start to diverge. There is no rise of the T_1_ to T_n_ peak, suggesting that acridine became protonated. From this time, the dynamics follows that of the pH 3 sample. The time scale of 30 ps is in remarkable agreement with the protonation time scale reported earlier for quinolone (28 ps), a lower molecular mass analog of acridine^7,9^. A similar time scale can be extracted from the dynamics monitored at 550 nm (Fig. 3f), where the stimulated emission is observed at pH 3 in the protonated sample, seen as a nearly constant negative ΔOD signal. At pH 7 the signal is positive with dynamics initially similar to pH 13. However, an additional decay component arises ~30 ps resulting in a faster decrease of the ΔOD signal. Such change is consistent with the protonation of acridine and the ensuing increase in PL emission by the acridinium cation. Importantly, while the analysis of the transient absorption measurements of the CD-acridine sample is complicated by the contributions of the CDs themselves, a very similar protonation rate emerges from the transient trace plotted at 465 nm (see Supplementary Fig. 13). Therefore, the spectroscopic analysis provides evidence that the protonation time of acridine (free or adsorbed at the surface of CDs) at pH 7 is only ~30 ps. Remarkably, this time is comparable with electron transfer to co-catalyst in many inorganic semiconductor-based photocatalysts and faster than photogenerated hole transfer in such systems^48,49^. Consequently, owing to the photobasic effect, the proton transfer could no longer be a rate-limiting step in photocatalytic H_2_ generation.

### Photocatalytic H_2_ evolution

**Fig. 4:**
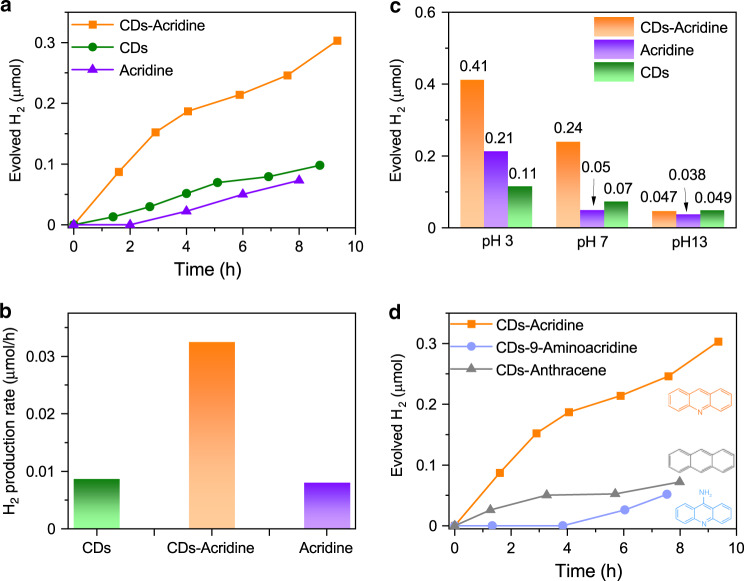
Photocatalytic hydrogen generation. **a** Hydrogen evolution and **b** the average hydrogen production rate for CDs, acridine, and the mixture under Xe lamp irradiation at pH 7; **c** the total amount of evolved hydrogen over 6 h of illumination. **d** Comparison of hydrogen generation of mixtures of CDs with acridine, a non-photobase (anthracene) and a photobase (9-aminoacridine) with pK_a_ 10.

In order to verify that the inclusion of a photobasic moiety affects the H_2_ generation rate, we illuminated the samples with a Xenon lamp and measured the evolved hydrogen (see Methods section for details). Methanol was used as a hole scavenger. As shown in Fig. 4, the rate of H_2_ generation at pH 7 is indeed much faster for the combined CD-acridine sample (0.032 μmol h^−1^) than for CDs only (0.08 μmol h^−1^) or acridine itself (0.08 μmol h^−1^). The increase in the rate is also obvious when the amount of H_2_ is normalized by the mass of either the CDs or of acridine (Supplementary Fig. 14). The increase is observed irrespective of the choice of the preparation method of the CD-acridine. In fact, it is even stronger (10-fold) for the sample where acridine is partially embedded inside the CDs (Supplementary Fig. 15). This implies that the stronger interaction between CDs and acridine, expected in this architecture, is indeed beneficial for the enhancement of H_2_ production. Furthermore, the increase is the strongest at neutral conditions where the photobase effect is expected to manifest itself (cf. Fig. 4c). In the alkaline conditions the amount of evolved H_2_ is the same as for the components individually. At low pH the increase can also be observed but is much less pronounced. This is to be expected, as at high proton concentration, its transport is unlikely to be a limiting factor. The fact that the rate of H_2_ generation is highest at pH 3 also indicates that proton transport could be limiting the process at its lower concentrations. In a control experiment, CD-acridine sample was illuminated through a 400-nm long-pass filter which cuts off the acridine absorption and leaves only the tail of CD absorption. The very small observed H_2_ production confirms that the absorption by acridine is needed for an improvement. Two further control experiments were performed to check whether just the adsorption of an acridine-like compound has an effect. To this end, we have added two structurally similar molecules to CDs which, however, do not exhibit photobasic behavior at pH 7: anthracene and 9-aminoacridine. The former is not a photobase at all, the latter has pK_a_ = 10 and even higher pK_a_*, so it cannot be protonated at pH 7^50^. In neither of these cases any increase in the H_2_ generation rate is observed (see Fig. 4d). This confirms that the photobasic property is critical to the enhancement. Finally, it is also important to establish the source of the evolved hydrogen. To this end, we performed a photocatalytic experiment in D_2_O instead of H_2_O. We noted a decrease in hydrogen generation rate by a factor of 1.43 (cf. Supplementary Fig. 16). This result implies a strong kinetic isotope effect and is consistent with water, rather than CDs or acridine, as the source of protons for the produced hydrogen^51^.

## Discussion

The rate of proton transfer determined by the time-resolved absorption measurements is consistent with the literature results obtained for smaller N-heterocyclic photobases (e.g. quinoline)^7,9^ and for acridine derivatives (e.g. acridine orange)^39^. Several studies on photobase – proton donor (water and alcohols) systems also report that the proton transfer can be accompanied or quickly followed by electron transfer from the same molecule^39,52,53^. The concurrent electron transfer might also explain why the protonation rate is faster than reported earlier for acridine^42,54^. It may facilitate a charge transfer complex pathway which is usually involved in picoscond timescale protonation processes^52,55^. Eventually, the resulting radical (e.g. Acr-H^•^) can undergo light-driven dissociation and lead to molecular hydrogen via Heyrovsky mechanism^56,57^. Acridine can only attract one proton, but in larger polycyclic structures with multiple photobasic moieties potentially in vicinity, Tafel reaction to form H_2_ may also be possible^58^. Here, in the CD-acridine system, there is second feasible source of the electron, i.e., the photoexcited CDs. The cyclic voltammetry measurements provide evidence that the band alignment is appropriate for the electron transfer from CD to acridine. It is anticipated that the electron transfer would be even more efficient for a photobasic moiety integrated (or embedded) in the aromatic domains of the CDs, that was postulated to occur in N-doped CDs^23^, and concurred by our experiments on the efficiency of the CDs with embedded acridine. The system comprising a CD and a photobase can thus transfer the two participating particles, a proton and an electron, to the reaction site at approximately the same time from two different sources, as depicted in Fig. 1. Under such scenario, any of the components would not have to wait for another, as they are delivered when needed, upon photoexcitation. This reduces the chances of parasitic charge recombination that inhibits photocatalytic activity. The second advantage of the CD-photobase system over a direct electron transfer from the proton donor is a generation of oxidized form of the latter (e.g. hydroxyl or alcoxyl radicals) in the vicinity of the reduction site. These reactive species could induce a back reaction with the H atom, reversing the benefit of the process. In a combined CD-photobase system, the photogenerated hole in the CD can be taken away by the scavenger at a more distant site, elsewhere on the dot. In such circumstances, the back reaction would be less likely. Moreover, it is worth noting that, while the experiments in the paper have focused on H_2_ generation, the proton transfer is an integral part also of the CO_2_ photocatalytic reduction. Thus, it can be surmised that the ability to induce the proton transfer via an increased basicity in the excited state could also be exploited in this process for increasing efficiency or selectivity of the CO_2_ reduction pathways.

In summary, we have shown that the proton transport in photocatalytic systems can be controlled by light. In the excited state photobase molecules can abstract a proton from the water within tens of picoseconds. This enables very fast - concerted or in quick succession - proton-coupled electron transfer and thereby substantially increases the rate of the overall photocatalytic process. We demonstrate this effect by fabricating nitrogen-free CDs and then including a model photobase, acridine, in the photocatalyst. This increases 4–10-fold the H_2_ generation rate of the sample in the neutral conditions. As photobasic moieties are building blocks of many photocatalytically active nitrogen-containing CDs and of carbon nitrides, we propose that the photobasic effect may be an important part of the mechanism of the process on these materials and may partially account for their success in the photocatalytic H_2_ production. We believe that the uncovering of this mechanism could contribute to an improved design of the carbon nanomaterial-based photocatalysts and lead to further enhancements in activity.

## Methods

### Materials

Polyethylene glycol (PEG, average Mn 400 and 600, ACS reagent ≥99.5%), ethanol, methanol, acridine, anthracene, 9-aminoacridine, and deuterium oxide (99.9% atom D) were purchased from Sigma Aldrich and used without further purification. Distilled water (ultrapure) was used for the synthesis and for diluting samples for characterization. Methanol was used as a hole scavenger in the photocatalysis experiments.

### Synthesis of CDs

The nitrogen-free CDs were synthesized by a straightforward pyrolysis method. Briefly, 318 mg of waxy solid PEG600 was dissolved in 10 mL ethanol. Then the transparent solution was transferred into a 15-mL poly(para-phenol)-lined stainless-steel autoclave and heated to 200 °C for 2 h. Afterwards, it was cooled down naturally to room temperature. The obtained solution was purified by centrifugation at 5000 rpm for 5 min with only the supernatant taken for further characterization. To correlate the optical properties of CDs with photocatalytic activity in water, ethanol was removed by evaporation in N_2_ flow and the sample was re-dispersed in the same amount of distilled water.

CDs-Acridine samples were prepared by two methods. In the first method, the aqueous solution of CDs was mixed with the saturated acridine solution in water in 1:3 (v/v) ratio, and then stirred with a magnetic bar to obtain a homogeneous solution. In the second method, acridine (same amount as in the first method) was mixed with PEG 400 in ethanol. This mixture was subsequently used as a reactant instead if pure PEG in the procedure that was the same as in CDs synthesis, described above. Ethanol in the sample was also removed and replaced with same amount of distilled water.

### Morphological and optical characterizations

For electron microscopy measurements, the samples were first centrifuged at 5000 rot min^−1^ for 5 min. Then an aliquot of the supernatant was drop-cast on a carbon coated copper grid and allowed to dry in the ambient environment. The transmission electron microscopy (TEM) and high-resolution transmission electron microscopic (HR-TEM) images were taken by a Titan Themis at 120 kV accelerating voltage. UV-vis absorption measurements were carried out with a Varian Cary 5000 UV-Vis-IR spectrometer. A Horiba Jobin Yvon Fluorolog-3 FL3-22 spectrometer with a 450W Xe lamp was used to acquire the photoluminescence spectra. Its double monochromators for both excitation and emission were mounted at 90° angle with a water-cooled photomultiplier tube. The resulting spectra were calibrated with excitation intensities and corrected. To perform time-resolved fluorescence measurements, a home-made device with a white light laser (NKT SuperK Extreme EXR-20), extended with an EXTEND-UV box, was used. The diluted samples were excited with 350 nm laser with a repetition rate of 5.56 MHz. The signal was detected with a Princeton Instruments monochromator fiber connected to a low-noise Avalanche Photodiode from Excelitas.

### Femtosecond transient absorption spectroscopy

Ultrafast transient absorption measurements in the present investigation has been conducted in a custom-built transient absorption setup from Newport. A multi-pass Ti: Sapphire femtosecond regenerative amplifier laser system (Libra HE+ from Coherent Inc.) produces ~100 fs laser pulse having 800-nm central wavelength and at 1 kHz repetition rate. A fraction of the 800-nm beam was passed through a spectrally tunable optical parametric amplifier (OPA) to produce the pump pulses at 350 nm. The near UV-visible probe pulses (300–750 nm) were generated by focusing another part of the initial 800-nm beam through a 2-mm-thick motorized CaF_2_ crystal. Further, the near UV-visible beam is split to two beams, probe and reference, to achieve better signal-to-noise ratios. The pump pulses were overlapped spatially and temporally on the sample with the probe pulses after passing through a 0.5-kHz optical chopper to read out the changes in probe transmission for each pump-probe cycle. To provide a time delay between the two pulses, a motorized optical delay line was positioned in the probe arm. MS260i spectrograph by Newport, Inc. acquired the transmitted signal used for further analysis of the data. Finally, the changes in the absorptions (ΔA) of the excited state were computed by subtracting the absorptions of excited and unexcited samples.

### Photocatalytic hydrogen generation

The experiments were conducted in a custom-built metal-free quartz cuvette. The 4.4 ml cuvette was filled with 2 ml aqueous solution containing 10% (v/v) methanol and placed 35 cm from the illumination source. A 450W Xe lamp was used for broad-band UV-Vis illumination without any filter, but as shown in the emission spectrum (Supplementary Fig. 17), the intensity decreases significantly for wavelengths shorter than 350 nm (10–15 mW intensity measured with 320 nm fluorescence filter). Prior to illumination, the cuvette was purged with argon for 1 min to remove air completely. The calculated illumination area was 1 cm^2^. The generated hydrogen was detected with a Shimadzu GC 2014 gas chromatograph by taking 10 μL aliquots of the headspace from the cuvette at regular time intervals and injecting into the GC. Argon was used as a carrier gas in the chromatograph.

## Supplementary information

Supplementary Information

## Data Availability

Data is available from the corresponding author upon reasonable request.
