# Supplementary material for: Photobase effect for just-in-time delivery in photocatalytic hydrogen generation
Source: Nat Commun. 2020 Oct 14;11:5179. doi: 10.1038/s41467-020-18583-6 (PMC7560858; doi:10.1038/s41467-020-18583-6)
Supplement: Supplementary file 1 — Supplementary Information [file 41467_2020_18583_MOESM1_ESM.pdf]

Supplementary Figures

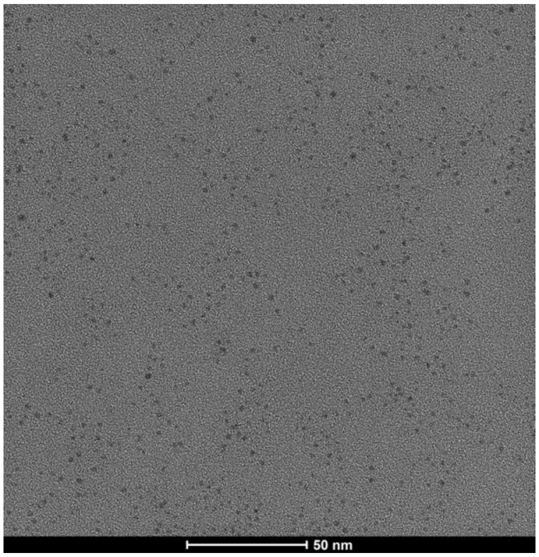

Supplementary Figure 1. Transmission microscopy. TEM image of the CDs.

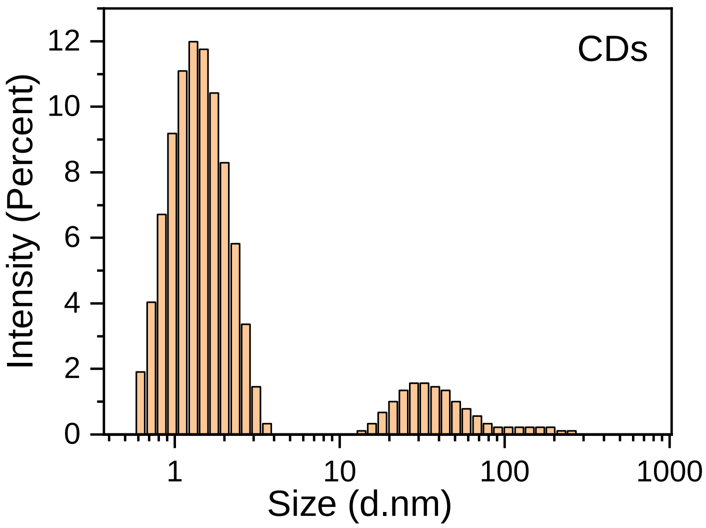

Supplementary Figure 2. CD size distribution. Intensity-weighted size distribution of the CDs obtained by dynamic light scattering.

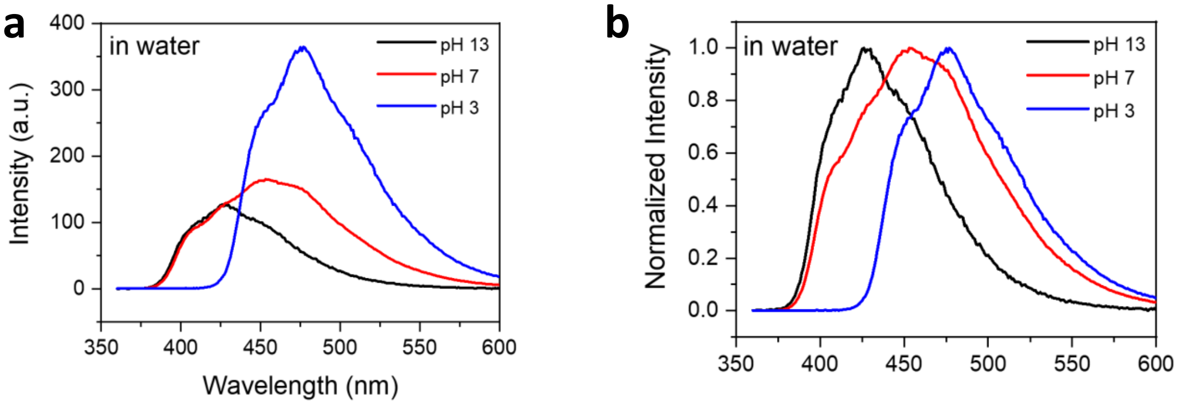

Supplementary Figure 3. Photoluminescence spectra of acridine. (a) Non-normalized and (b) normalized PL spectra of acridine in water at pH 3, 7 and 13. The integrated PL intensity at pH 7 is higher than at pH 13 by a factor of 1.77. The intensity at pH 3 is higher than at pH 7 by a similar factor, 1.73. Comparing the intensities, it is clear that only 3% of protonated acridine (percentage of protonated acridine at pH 7) would not account for the whole increase between pH 13 and 7. This control experiment provides evidence that some non-protonated acridine molecules when excited at pH 7, become protonated and emit as the acridinium cation.

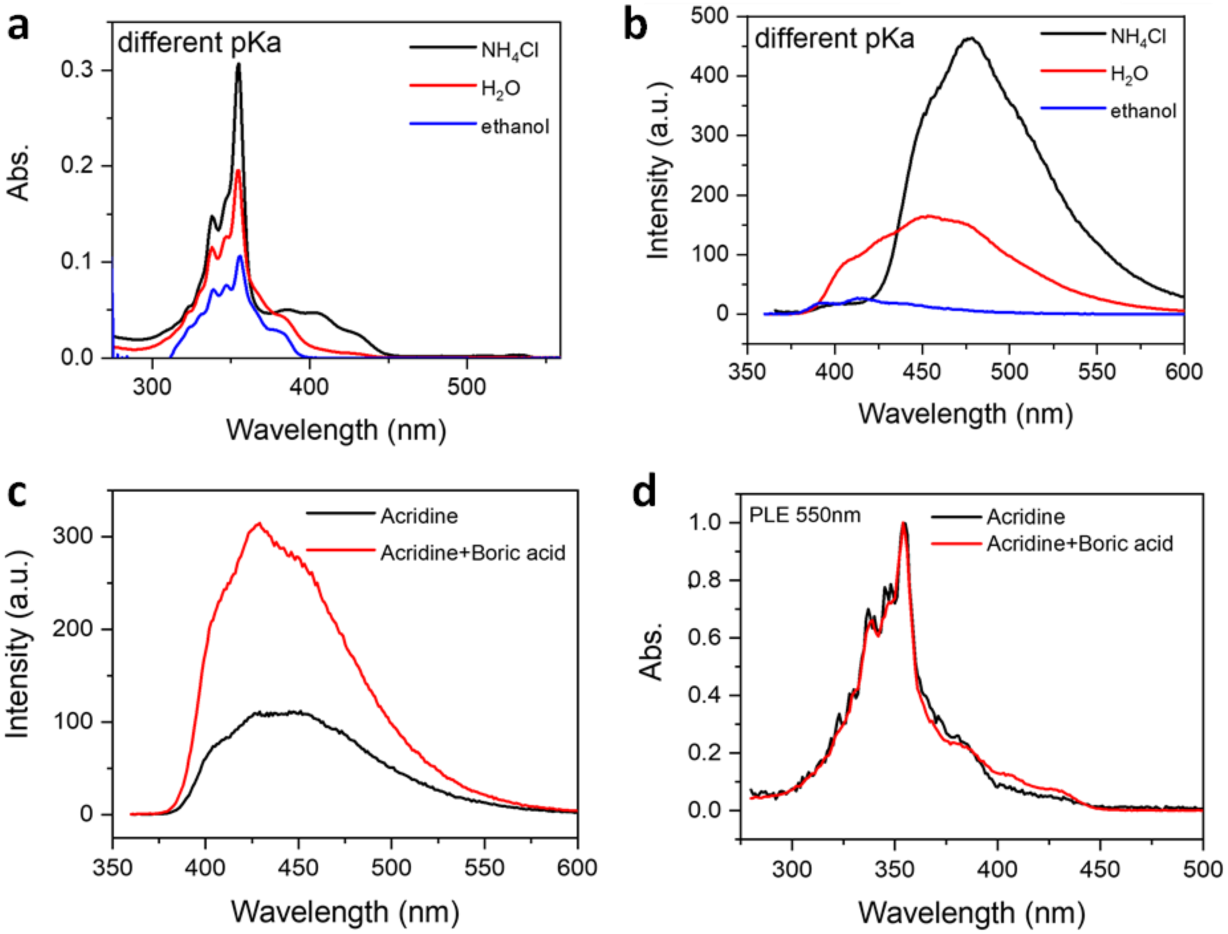

Supplementary Figure 4. Spectroscopic analysis of protonation of acridine. Comparison of (a) absorption and (b) emission spectra of acridine in ethanol, water and aqueous solution of NH_4Cl, taken at the same concentration of acridine; (c) PL and (d) PLE spectra of acridine in water with and without addition of boric acid. Ethanol leads to a decrease in PL intensity. On the other hand, the PL intensity increases in the NH_4Cl and boric acid, in line with the expected increased protonation. The PLE spectra with and without the boric acid are identical suggest that the non-protonated species are being excited from the ground state in both cases.

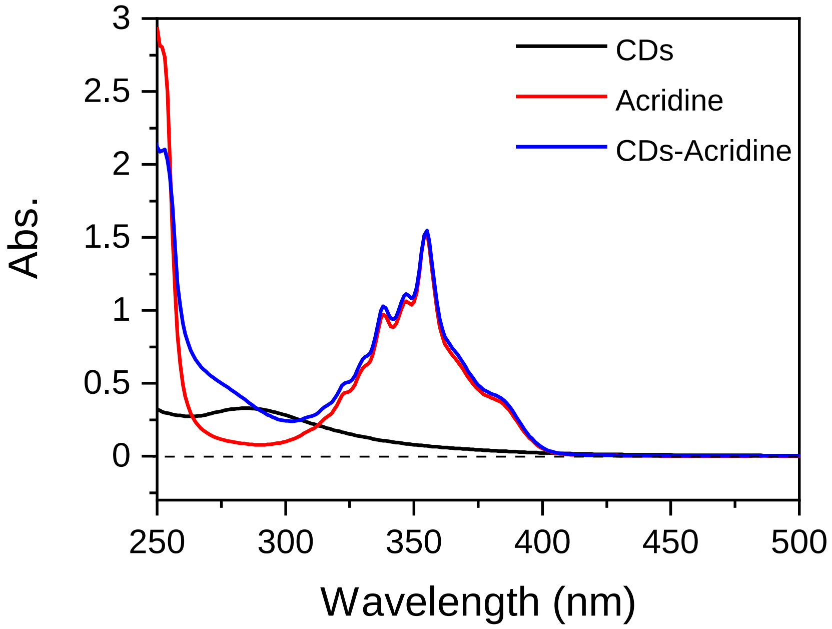

Supplementary Figure 5. Absorption spectra at pH 7. Comparison of the non-normalized absorption spectra of CDs, acridine and CD-acridine taken at pH 7.

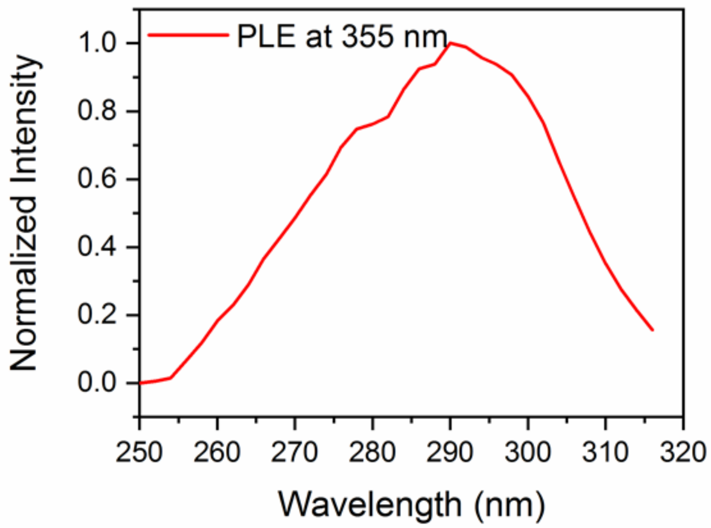

Supplementary Figure 6. Photoluminescence excitation spectrum at 355 nm. The PLE spectrum of the CD-acridine sample, measured at 355 nm.

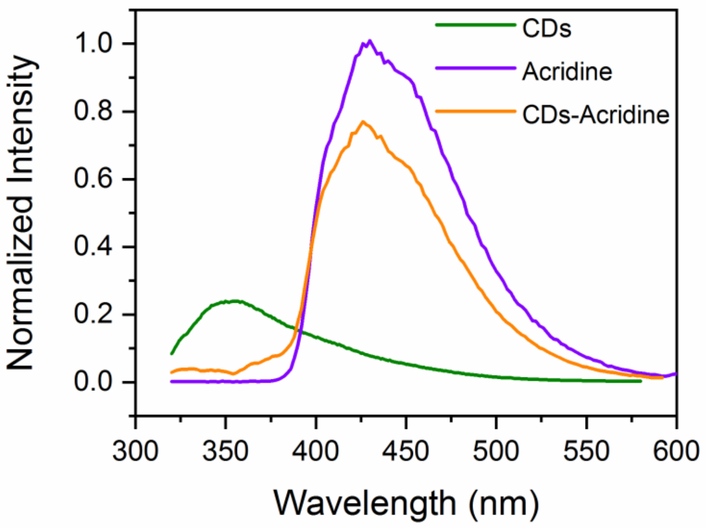

Supplementary Figure 7. Photoluminescence spectra. Comparison of the PL spectra of CDs, acridine and the CD-acridine sample acquired under 300 nm excitation.

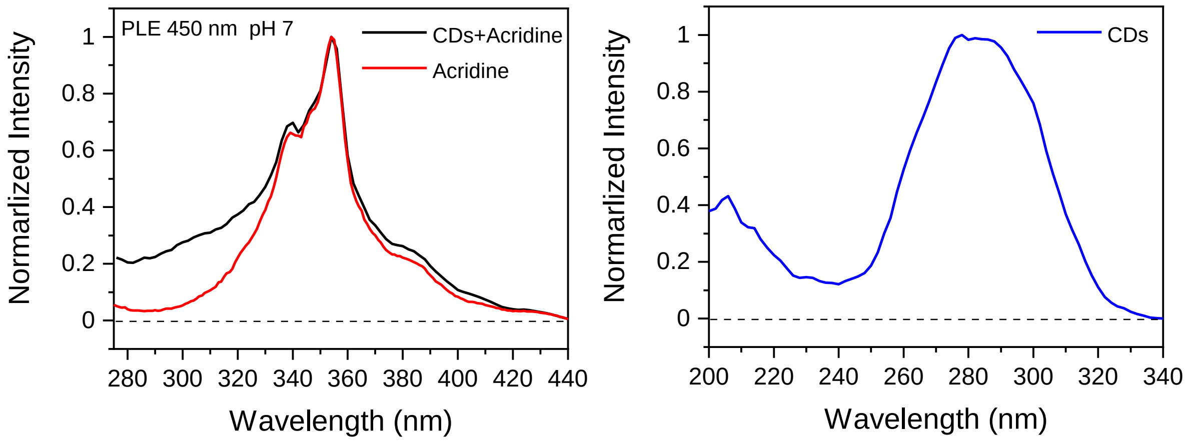

Supplementary Figure 8. Photoluminescence excitation spectrum at 450 nm. PLE spectra of (a) acridine and CD-acridine, and (b) CDs with the detection wavelength set to 450 nm.

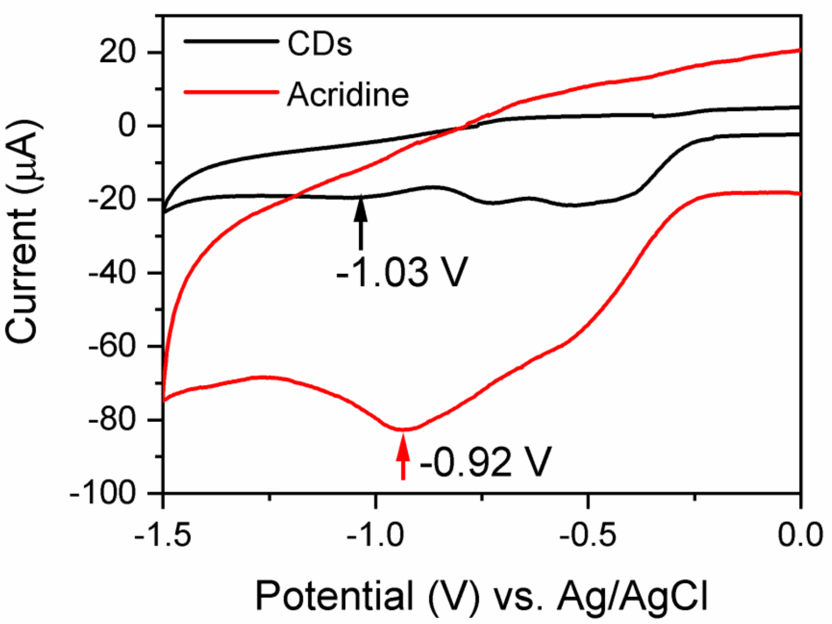

Supplementary Figure 9. Cyclic voltammetry. Cyclic voltammograms of aqueous solutions of CDs and of acridine acquired at the scan rate of 100 mV/s. The arrows indicate the positions of the reduction waves attributed to the conduction band potential of the CDs (black line and arrow) and LUMO level of acridine (red line and arrow). The details of the cyclic voltammetry measurements are given in the Supplementary Methods.

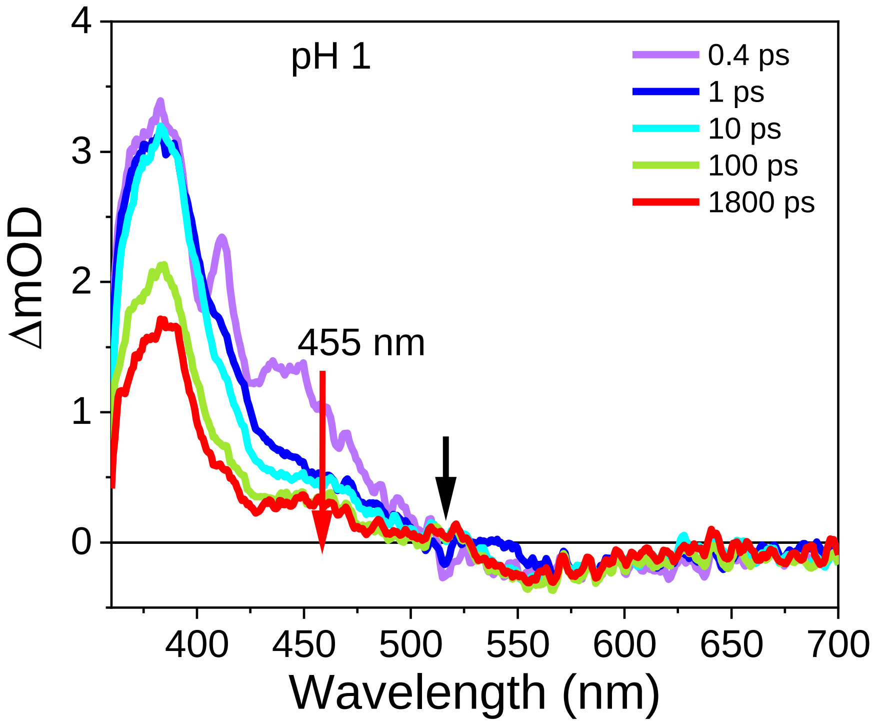

Supplementary Figure 10. Transient absorption spectra of acridine at pH 1. The black arrow indicates the expected position of the peak observed at 512-518 nm at higher pH. The red arrow at 445 nm indicates the ESA peak associated with the S_1 to S_n transition of the protonated acridine.

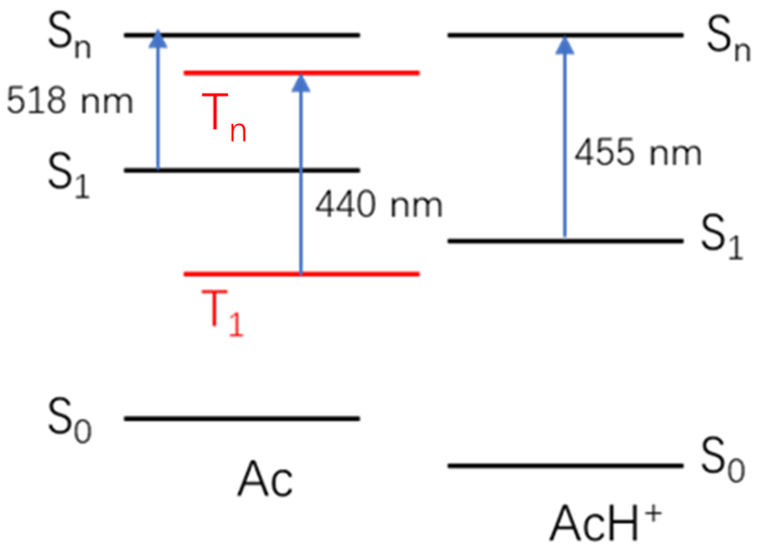

Supplementary Figure 11. Acridine energy diagram. Schematic energy diagram of (left) acridine and (right) acridinium cation.

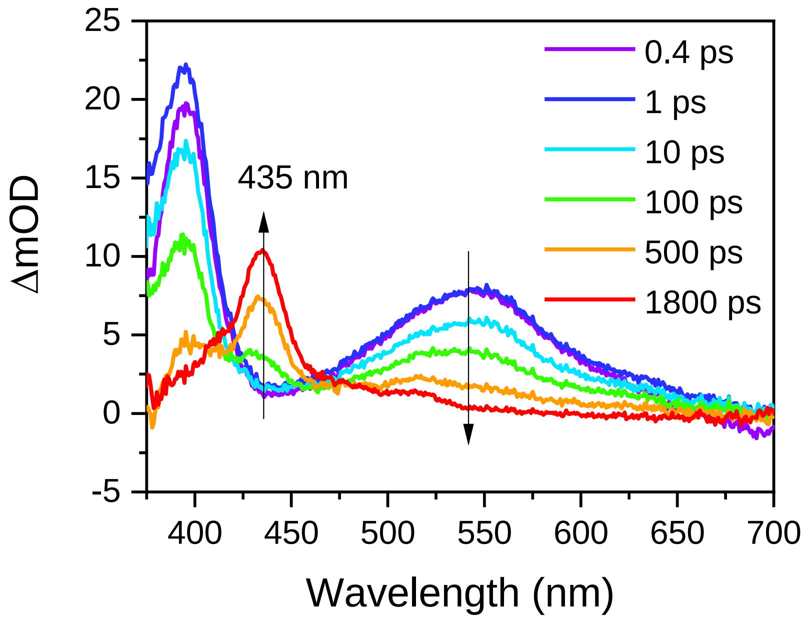

Supplementary Figure 12. Transient absorption spectra of acridine in ethanol. The arrows illustrate the decay of the excited state absorption (ESA) peak at 535 nm and the rise of the ESA peak at 435 nm.

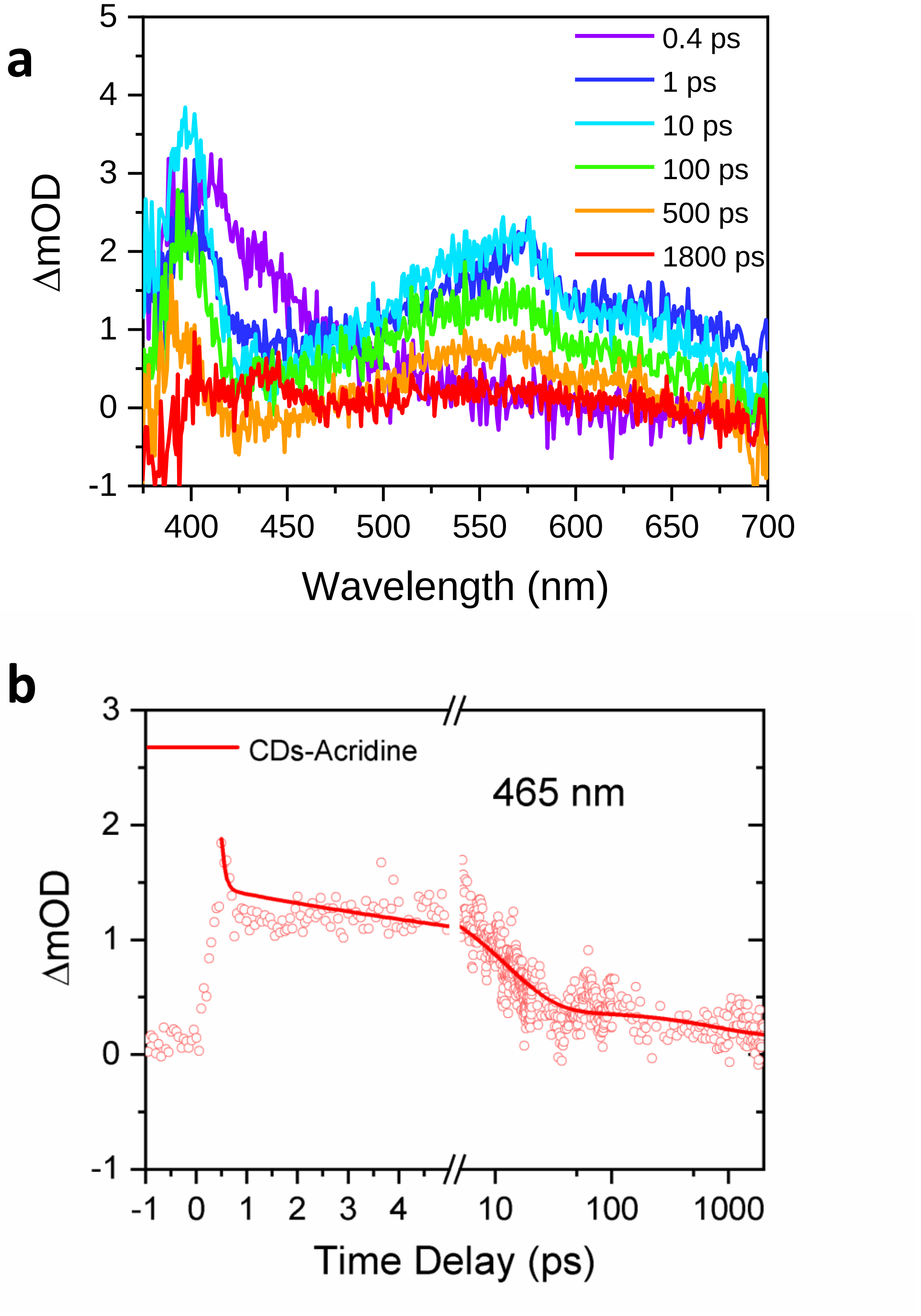

Supplementary Figure 13. Transient absorption spectroscopy of CD-acridine. (a) Transient absorption spectra of the aqueous dispersion of the CD-acridine sample taken at pH 7; (b) Transient absorption trace of the sample at 465 nm. The open circles represent the data points, the solid line corresponds to a multiexponential fit, plotted as guide for the eye. The details of the fitting procedure are given in the Supplementary Methods.

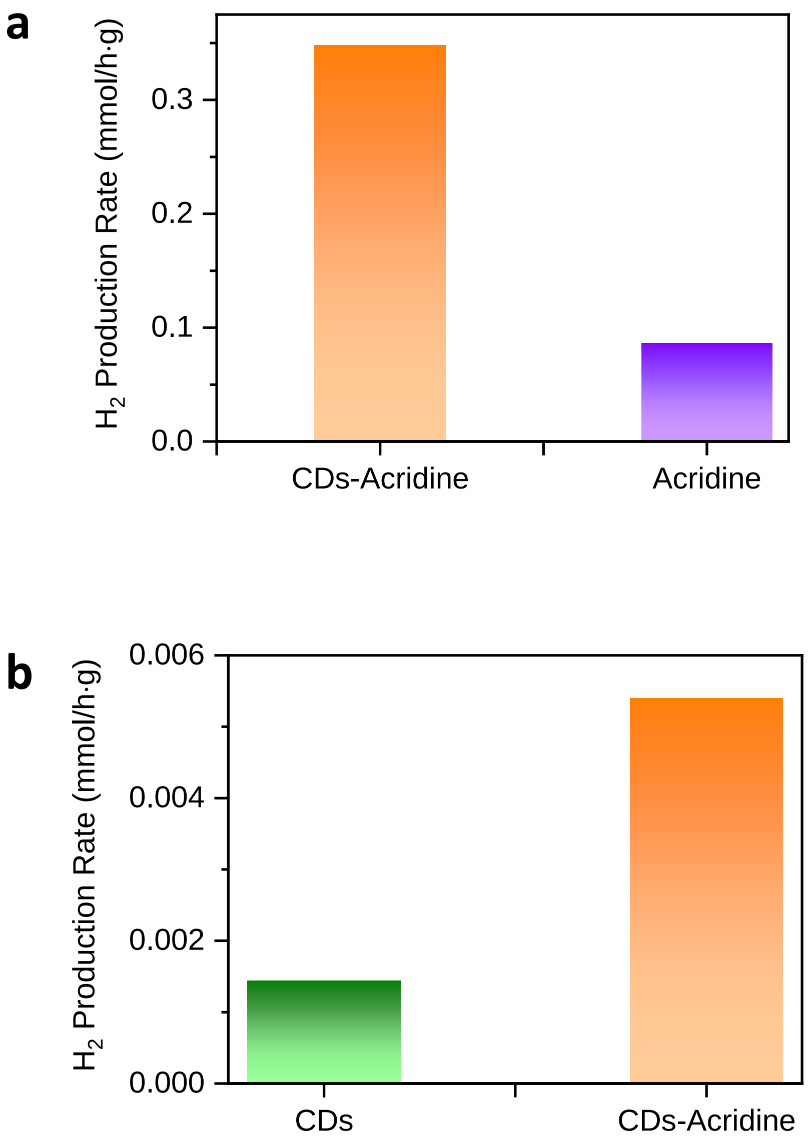

Supplementary Figure 14: **Photocatalytic H_2 generation experiments.** Photocatalytic hydrogen generation rate normalized by (a) mass of acridine and (b) mass of CDs, respectively.

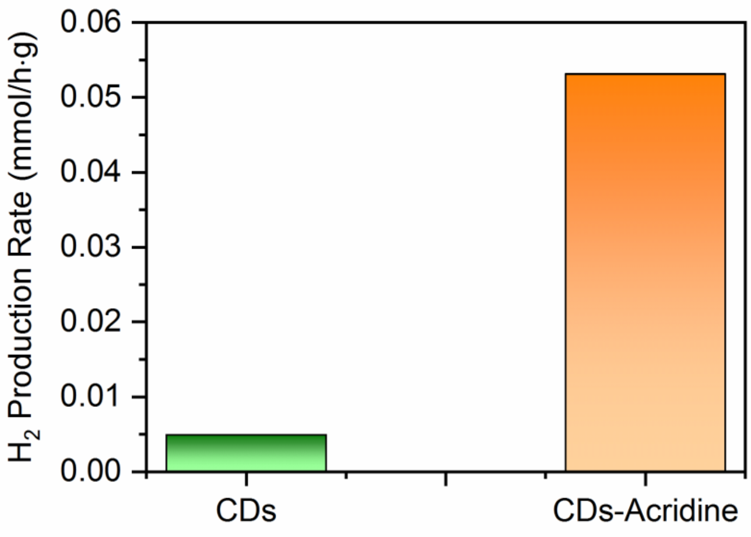

Supplementary Figure 15: **Photocatalytic effect of acridine embedded in CDs.** Photocatalytic hydrogen generation rate of CDs-Acridine synthesized by second method (acridine added during the synthesis of CDs from PEG), normalized by mass of CDs.

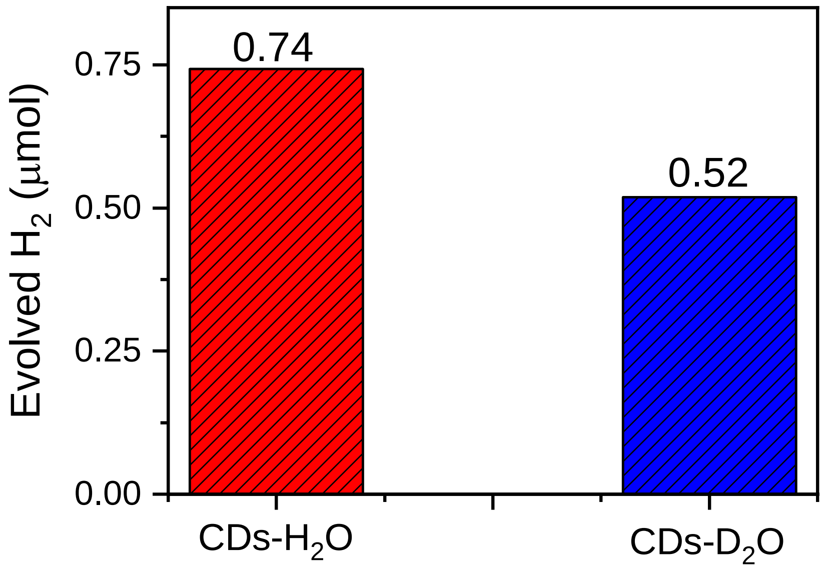

Supplementary Figure 16. Isotope effect. Comparison of hydrogen generation in H_2O and D_2O.

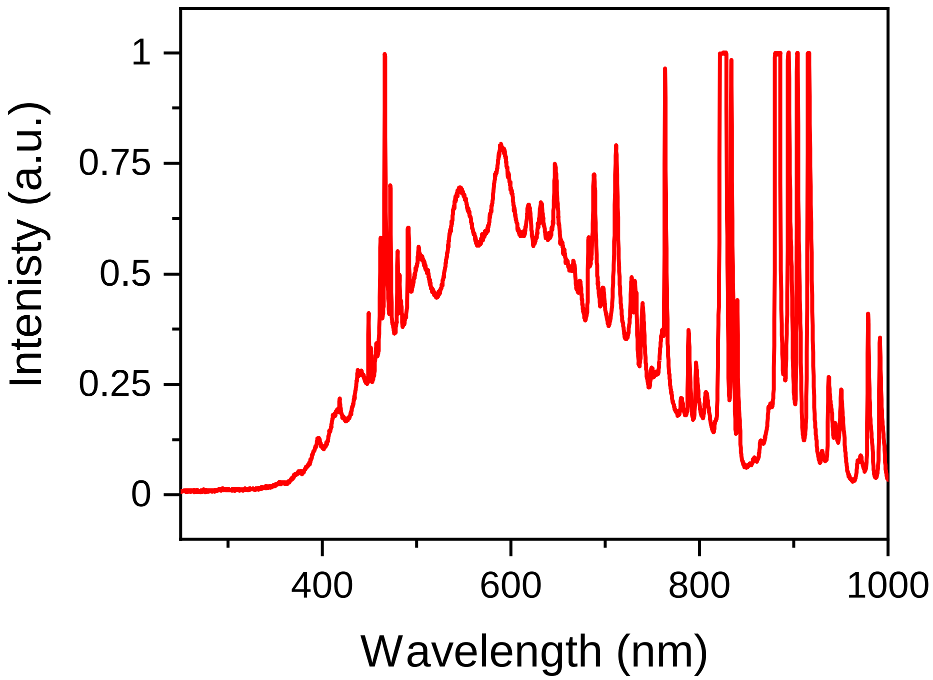

Supplementary Figure 17. Xe lamp emission spectrum. The emission spectrum of the xenon lamp used in the photocatalytic experiments.

Supplementary Tables

Supplementary Table 1. Transient optical absorption decay kinetics fit at 440 nm to a sum of two or three exponential terms.

pH value	τ_1 (ps)	τ_2 (ps)	τ_3 (ps)
3	0.125	4.59	
7	0.103	35.3	-935.5
13	0.125	53.27	-758.2

The negative values of τ_3 for pH 7 and pH 13 represent a growth term, rather than a decay term. This corresponds to a triplet state formation.

Supplementary Table 2. Transient optical absorption decay kinetics fit at 465 nm to a sum of two or three exponential terms.

pH value	τ_1 (ps)	τ_2 (ps)	τ_3 (ps)
3	0.099	7.30	
7	0.163	29.69	
13	0.123	60.97	-513.9

The negative value of τ_3 for pH 13 represents a growth term, rather than a decay term. This corresponds to a triplet state formation.

Supplementary Table 3. Transient optical absorption decay kinetics fit at 550 nm to a sum of two or three exponential terms.

pH value	τ_1 (ps)	τ_2 (ps)	τ_3 (ps)
3	0.241	6.14	
7	0.24	30	1617
13	0.14	12.2	

Supplementary Notes

Supplementary Note 1: Acridine protonation equilibria

The reaction of water deprotonation by acridine is given by:

$$Ac + H_2O \rightleftarrows AcH^+ + OH^- \quad (1)$$

The equilibrium constant is then given by the Supplementary Equation 2:

$$K = \frac{[AcH^+][OH^-]}{[Ac]} \quad (2)$$

The reaction of dissociation of acridine conjugated acid can be written as:

$$AcH^+ \rightleftarrows Ac + H^+ \quad (3)$$

With the equilibrium constant $K_{a,Ac}$:

$$K_{a,Ac} = \frac{[Ac][H^+]}{[AcH^+]} \quad (4)$$

pK_a of acridine in the ground state is 5.5, in the excited state it rises to 10.7. For water the dissociation can be written as

$$H_2O \rightleftarrows H^+ + OH^- \quad (5)$$

with $K_{a,water}$ defined as

$$K_{a,water} = [H^+][OH^-] \quad (6)$$

pK_a of water equals 14, so that $K_{a,water} = 10^{-14}$. Combining the two reactions, K can be calculated from the Supplementary Equation 7:

$$K = \frac{K_{a,water}}{K_{a,Ac}} \quad (7)$$

Hence, in the ground state of acridine, K equals $10^{-(14-5.5)}$ = 3.2·10-9. In the excited state, K equals $10^{-(14-10.7)} = 5.0 \cdot 10^{-4}$. From this the ratios of the concentrations of the protonated to deprotonated acridine can be calculated for any given pH:

$$\frac{[AcH^+]}{[Ac]} = \frac{K}{[OH^-]} \quad (8)$$

For instance, at pH 7 the ratio in the ground state is 0.032, so that around 3% of acridine molecules are protonated. In the excited state the ratio is ~5000, so that the majority of the acridine molecules are protonated.

Additionally, it should be noted that the deprotonation of water in a closed volume leads to the formation of OH^- anions that would increase the pH and decrease the protonation ratio. The upper limit of this effect is given by the concentration of the saturated solution of acridine in water which is only 0.25mM. Assuming all acridine molecules are excited, their protonation would produce 0.25mM of OH^-, that is pH 10.3. This is still below the pK_a of excited acridine, so that more than 50% of excited acridine molecules should be in the protonated form. In practice, this assumption is unrealistic, fewer acridine molecules would be excited, the pH shift would be smaller and the ratio of excited protonated acridine to excited non-protonated acridine closer the one calculated above for pH 7.

Supplementary Methods

Fitting procedure for TCSPC data:

All TCSPC decay curves were fitted with biexponenital decay models given by the Supplementary Equation 9:

$$I = A_1 e^{\frac{-t}{\tau 1}} + A_1 e^{\frac{-t}{\tau 2}} + I_0 \tag{9}$$

Where I is the normalized PL intensity; A_i (i=1, 2) and τ_i (i=1, 2) are the fractions and lifetimes of the two decay components, respectively. I_0 is a baseline constant. The average lifetime (τ_{avg}) was calculated according to the Supplementary Equation 10:

$$\tau_{\text{avg}} = \frac{\sum A_i \tau_i^2}{\sum A_i \tau_i} \tag{10}$$

Fitting procedure for transient absorption data:

The transient absorption traces have been fitted with multi-exponential functions according to the Supplementary Equation 11 using the Origin software:

$$y = y_0 + A_1*\exp(-x/\tau_1) + A_2*\exp(-x/\tau_2) + \ldots \tag{11}$$

Here τ_1, τ_2 … are the fitted lifetime of different components and A_1, A_2… are corresponding amplitudes. For all the traces, there is a pulse-width limited rise in the transient absorption signal and therefore, 0.2 ps to 2 ns fitting range have been used. The Supplementary Tables 1-3 show the fitted time constants for different transient absorption traces for acridine at different pH values for the traces recorded at 440 nm, 465 nm and 550 nm, respectively.

Cyclic voltammetry:

Autolab N series Potentiostat/Galvanostat was used for the CV measurements, together with a standard three-electrode system with a commercial glassy carbon (CHI instrument, USA, 5.61 mm diameter) working electrode, Pt wire counterelectrode, and Ag/AgCl reference electrode. The pH of the deionized water was adjusted to 9 with sodium hydroxide to enhance the conductivity. Typically, 5 mg of the sample and 100 μL 5 wt% Nafion solution were dispersed in 1 mL water/isopropanol (3/1, v/v) solution and ultrasonicated for 30 min to form homogeneous slurry. Next, 4.5 μL of the slurry was drop-cast on the polished working electrode and dried in the room temperature. The electrodes were then immersed in the electrolyte for 20 minutes to reach equilibrium state. The cyclic voltammograms were acquired between -1.5 V to 1.5 V with a scan rate of 100 mV/s.
